# Adult *Alphitobius diaperinus* Microbial Community during Broiler Production and in Spent Litter after Stockpiling

**DOI:** 10.3390/microorganisms10010175

**Published:** 2022-01-14

**Authors:** Tawni L. Crippen, Baneshwar Singh, Robin C. Anderson, Cynthia L. Sheffield

**Affiliations:** 1 Southern Plains Agricultural Research Center, Agricultural Research Service, United States Department of Agriculture, College Station, TX 77845, USA; robin.anderson@usda.gov (R.C.A.); cindy.sheffield@usda.gov (C.L.S.); 2 Department of Forensic Science, Virginia Commonwealth University, Richmond, VA 23298, USA; bsingh@vcu.edu

**Keywords:** darkling beetle, chicken litter, microbiome

## Abstract

The facilities used to raise broiler chickens are often infested with litter beetles (lesser mealworm, *Alphitobius diaperinus*). These beetles have been studied for their carriage of pathogenic microbes; however, a more comprehensive microbiome study on these arthropods is lacking. This study investigated their microbial community in a longitudinal study throughout 2.5 years of poultry production and after the spent litter, containing the mealworms, was piled in pastureland for use as fertilizer. The mean most abundant phyla harbored by the beetles in house were the Proteobacteria (39.8%), then Firmicutes (30.8%), Actinobacteria (21.1%), Tenericutes (5.1%), and Bacteroidetes (1.6%). The community showed a modest decrease in Firmicutes and increase in Proteobacteria over successive flock rotations. The beetles were relocated within the spent litter to pastureland, where they were found at least 19 weeks later. Over time in the pastureland, their microbial profile underwent a large decrease in the percent of Firmicutes (20.5%). The lesser mealworm showed an ability to survive long-term in the open environment within the spent litter, where their microbiome should be further assessed to both reduce the risk of transferring harmful bacteria, as well as to enhance their contribution when the litter is used as a fertilizer.

## 1. Introduction

The phylum Arthropoda is the most abundant and diverse clade of animals [1,2]. The microbes that they harbor either externally or within their gut microbiome are ecologically important as beneficial or pathogenic and are key facilitators of the varied lifestyles of their arthropod hosts. In general, the bacterial phyla Proteobacteria and Firmicutes predominate in the alimentary canal of most insect species even encompassing different general feeding strategies; however, at the lower taxa levels (i.e., class, order), the bacterial community composition varies widely [3,4]. Not unexpectedly, the community structure is influenced by diet and host taxonomy, and is also altered by the developmental stage, the host niche environment, transient environmental factors, and other biotic and abiotic factors [3,4,5].

The lesser mealworm (LM), *Alphitobius diaperinus* (Panzer) (Coleoptera: Tenebrionidae), is being explored for commercial production of insect protein feed, as well as utilization of their frass for fertilizer, and as a possible environmentally friendly method to degrade plastics [6,7]. However, in stored grain products and in the litter and manure pits of poultry operations, it is a pest. This arthropod is thought to be native to sub-Saharan Africa, but has been found throughout the world for so long that its actual origins are uncertain. It is a tropical species, so it does well in warm, humid environments such as caves, but unfortunately in grain silos, and poultry houses, as well. Broiler litter contains many components (bedding, feed, insects, feathers, carcasses, etc.) that have beneficial nutrients, including nearly 30% crude protein, along with minerals and some heavy metals and is considered a valuable commodity for secondary use as a fertilizer once it can no longer sustain broiler chickens [8,9]. Interestingly, the LM population within litter can be quite dense and the insects themselves are composed of a high percentage of protein, as well as variable microbes, both beneficial and problematic [10,11,12]. However, the contribution of the microbial community within the litter from the LM is not usually considered separately. While many studies have explored the presence of viral and bacterial pathogens harbored by these beetles [13], to date little has been done to explore its entire microbiome. In its manmade setting, as they inhabit the litter of poultry production facilities, they can be inadvertently transferred when litter is subsequently used as fertilizer for crops, gardens, and pastureland. Arunraj et al. [14] demonstrated that LM in poultry litter transported and placed in open fields did not die, but dispersed and hid in the local environment. So, it is important to consider the microbiome that comes with these arthropods both for its affects within the poultry house and as it is unintentionally redistributed into the environment.

The research reported here was designed to determine the microbiome of adult LM found within the poultry litter from a newly constructed house through 11 flock rotations (2.5 years) and after beetles were transported into pastureland within spent litter used as fertilizer. The focus was on how poultry management practices affect retention or reduction of the bacteria that comprise the beetle microbiome while in the house and the composition of microbes that could be redistributed into the environment after deposition with spent litter as fertilizer.

## 2. Materials and Methods

### 2.1. Site Description

The new temperature controlled broiler production house was constructed on an open range Post Oak Savannah facility in NW Robertson County, Texas [15]. The soil underneath was a fine, smectitic, thermic Udertic Paleustalfs with slopes ranging from zero to three percent (USDA-NRCS Official Soil Series Description available at https://soilseries.sc.egov.usda.gov/osdname.aspx (accessed on 9 December 2021)). The samples were collected from February 2008 to August 2010 utilizing full personal protective equipment and aseptic technique. The collection spanned 11 flock rotations in duration. The broiler facility is a standard dirt floored, tunnel-ventilated, metal house, 14 m in width (North/South) by 152.4 m in length (East/West). It was placed on 25 cm of commercial-grade clay-based topsoil and has alternating water and feed lines running the entire East/West length of the house (Figure S1).

### 2.2. In-House Management Practices

Fresh pine chip bedding was placed into the house to a depth of 15.3 cm (approximately 32 metric tons), prior to placement of the first flock of poults. Broiler chickens (Ross^®^ 708) were fed a corn/soy-based ration with 50 ppm bacitracin (Table S1). Each flock had a stocking density of one broiler per 0.1 m^2^ (25,800 birds per rotation). The 1 to 2 day old poults were placed and confined to half of the house for a 2 week period, before being allowed to access the entire house. The chickens were reared for 6 to 9 weeks (averaging 59 ± 6 days). The house was left empty an average of 11 days, between flock rotations. At this facility, the birds were healthy over the course of this study. For this study, litter is defined as bedding after use by the birds. The producer performed a partial house cleanout (PCO) prior to the 8th flock rotation, consisting of the removal of 5 to 8 cm of litter including the top caked layer followed by the addition of 6.4 cm of fresh bedding. Prior to the 10th flock rotation, a total house cleanout (TCO) was performed by removing of all litter plus a 1–3 cm of the pad-soil. Fresh bedding (15.3 cm) was then added.

### 2.3. Collections of Beetles within the Poultry House

After construction, the house was supplied with feeders, waterers and bedding. Birds were first placed in the house on the third week. For collection purposes, the house was divided into Side A and Side B. The first 2 weeks of each flock rotation, the birds were restricted to half of the house by a brooder curtain; Side A was left empty, and Side B contained the new flock of birds. The house was divided into 10 collections sites (Figure S1) and LM adult beetles were collected for this study. LM first populated the house along the inside perimeter of the building on week 7 (the end of Flock 1). Flocks 2 and 4–11 were sampled twice only on the first and final week of each flock rotation (Flock) within 24 h after bird placement or bird removal. Due to logistical issues with the producer and spanning the holidays, Flock 3 was not sampled. The beetles slowly spread toward the center of the structure, hiding under the feeders, until finally found throughout the house. Therefore, the beetle collections had to be strategized at each time point based on the beetle locations. Initially, 12 replicate 7.6 × 30.8 cm” polyvinyl chloride pipe tubing traps with rolled fluted corrugated cardboard inserts were placed along the perimeter of the house in areas 1, 5, 6, and 10 as shown in Figure S1. For collection, the corrugated insert was sterilely removed, and beetles shaken into a resealable plastic bag. This was continued for the 5 collection time points between weeks 7 and 42 (the end of Flock 4) (*n* = 60). By week 43 (the start of Flock 5), the beetles had spread from the perimeter to the interior of the structure and were collected from under the feeder stations along the 3 feeder lines: areas 2, 3, 4, 7, 8 and 9 as shown in Supplementary Materials Figure S1 covering each side (A or B). The interior samples were collected using sterile gloves and each sample was placed into an individual sterile zip-top bag. A total of 3 random sites within each of the 6 interior areas were sampled (*n* = 252) along with the 12 perimeter samples (*n* = 168) over the remaining 14 time points. All samples were transported from the field site at ambient temperature and processed the same day upon return to the laboratory. The beetles were separated from the litter with sterile forceps. The perimeter and interior samples were combined at the laboratory into 2 composite samples per Side A (areas 1, 2, 3, 4 and 5) and 2 composite samples per Side B (areas 6, 7, 8, 9 and 10) at each time point. A total of 480 samples were collected in house over the course of this study and combined into 76 composite samples for DNA analyses (In-House Beetle).

### 2.4. Collections of Beetles in Spent Litter

After Flock 9, the spent litter was removed from within the poultry house and transported to land used as pasture, that had not previously received litter supplement. In total approximately 30 km representing 5.5 truckloads (21 MT/truck) were moved. A composite native soil sample was taken from the location where the litter was to be piled. The litter was then placed in 6 contiguous rows ranging in height from 1.8 to 2 m and covering a surface area of 2100 m^2^. A single composite LM sample (Spent Beetle) was collected as described above, made from 10 random sites across the pile for each time point (0, 1, 2, 3, 5, 7, 9, 11, 13, 15, 17, and 19 weeks). This occurred from April until spreading in August at a rate of 0.9 MT/0.4 ha between weeks 19 and 20. As no LM could be found at the site 2 days after the spent litter was spread, only a combined soil/litter sample was taken (Post Spread) at that time. Weekly weather data were gathered from Weather Underground, an online service of The Weather Company, Inc., Atlanta, GA (Table S2). All samples were collected into sterile zip-top plastic bags and kept at ambient temperature during transport to the laboratory. Samples were processed the same day where the LM were separated from the litter with sterile forceps. A total of 120 LM samples were collected from the spent litter over the course of this study and combined into 12 composite samples for DNA analyses (Spent Beetle).

### 2.5. 16 S rDNA Analysis

At the laboratory, the beetles from each area were manually separated from their substrate using sterile forceps and an aliquot of 30 random beetles from each area were homogenized in lysing solution from FastDNA™ SPIN Kit (MP Biomedicals, Santa Ana, CA, USA) then incubated at 4 °C one hour before DNA was extracted per manufacturer instructions. The resulting DNA was quantified using Quant-iT™ PicoGreen™ dsDNA Assay Kit (ThermoFisher Scientific, Waltham, MA, USA) and a Wallac 1420 Victor 3™ fluorescent microplate reader (PerkinElmer, Altham, MA, USA). The DNA concentration was standardized to 20 ng/µL and samples were combined into 8 composite beetle samples (as described above) from within the house (In-House Beetle) and a single composite beetle sample from the spent litter (Spent Beetle), per time point. The composite samples were thoroughly mixed by slow rotation for 5 min and the resulting DNA extracts were sent overnight to the Research and Testing Laboratory, LLC. (Lubbock, TX, USA) and processed as previously described [16].

Sequence analyses were conducted as previously described [16]. Suspected chimeric sequences were deleted and 171,141 good-quality sequence reads were used for hierarchical classification as previously described [16]. All sequences not classified as bacteria at ≥80% bootstrap support (*n* = 1) were deleted. To avoid spurious OTU count due to the different number of sequence reads in different samples, to avoid spurious OTU count all sequences were subsampled to 576 reads for α- and β-diversity (Yue and Clayton at 0.03 genetic distance) estimations. Subsampling caused a loss of four In-House Beetle samples out of total 92 composite samples, from further analysis. Non-metric multidimensional scaling (NMDS) plots utilized Yue and Clayton distances in Mothur v 1.41.3 and the data were plotted using the rgl v 0.100.54 package in R (version 4.0.0) [17].Heat map graphics of the top 55 genera were generated using natural log-transformed percent relative sequence abundance profiles in the gplots v 3.0.3 package in R version 4.0.0 and in Prism 7 (Graph Pad, La Jolla, CA, USA). The 0% values were converted into 0.01% for log transformation. On the *X*-axis, bacterial genera were clustered by Unweighted Pair Group Method with Arithmetic Mean (UPGMA) based on weighted Unifrac distances. All trees were edited using FigTree v1.4.2 (http://tree.bio.ed.ac.uk/software, accessed on 9 December 2021). All raw sequence files were submitted to European Nucleotide Archive Database as part of this study (accession# PRJEB47980).

### 2.6. Statistical Analysis

No significant differences within flock rotations or between collection sites were found for In-House Beetle samples during initial analyses using molecular variance (AMOVA) (Yue and Clayton distance at 3% genetic dissimilarity). Therefore, the samples were combined as replicates based on flock rotation for further analysis. Indicator genera associated with each treatment were determined as previously described [16].

## 3. Results and Discussion

Microbial diversity and community changes were investigated within the LM population at a newly built poultry house. The collections were made throughout 11 Flock rotations, one partial cleanout (after Flock 7) and one total cleanout (after Flock 9). The low level of Bacitracin was used in the feed has some bactericidal activity on Gram-positive organisms and suppresses necrotizing enteritis in poultry (https://www.merckvetmanual.com/poultry/necrotic-enteritis/overview-of-necrotic-enteritis-in-poultry; accessed on 9 December 2021) and could have effects on the microbial diversity within the beetles in the house. The beetles were homogenized prior to analysis; therefore, the results indicate the microbial community of the whole insect. It is recognized that the sequencing platform only sequences small fragments, which is less precise when trying to identify bacteria at the genus/species level.

### 3.1. In-House Beetles

Beetles were sampled from when they first appeared in this newly constructed broiler house near the end of Flock 1 through 11 flock rotations.

#### 3.1.1. In-House Beetles: Alpha Diversity

The bacterial taxonomic diversity in the house LM between the successive flock rotations was investigated (Table 1). Rarefaction curves are shown in Figure S2. The indices in Table 1 provide information about the rarity and commonness of species present in the bacterial communities harbored by LM. In considering both the number of unique microorganisms (richness) and relative abundance of different species present (evenness), with emphasis on the evenness component, the Inverse Simpsons index measured higher values for Flock 5 beetles indicating it as having the most diverse community. Flock 2 beetles had the lowest diversity of microbial organisms. The Shannon index, which emphasizes the richness, also indicated that Flock 5 beetles had the most diverse and Flock 2 beetles had the least diverse microbial population.

Within the broiler house, the influence of the management practices is of interest in terms of the retention of beneficial and harmful microbes by the LM adult population. Interestingly after the partial and total cleanouts, the addition of new bedding for Flocks 8 and 10, resulted in no significant measurable changes in the LM microbial diversity. Denoting that this management practice had little effect the microbial community harbored by In-House LM; however, this might not be the case in all broiler houses. Many factors influence the microbial community within each broiler house, such as the feed ration used, breed and age of birds reared, the spillage of feed or water, the bedding type, the number of birds stocked per house, and the depth and timing of the poultry house clean-out practices [18]. These factors vary by production facility and their standard operating procedures that conform to the particular industry for which the birds are being produced.

#### 3.1.2. In-House Beetle: Indicator Species

Apparent bacterial indicator species for the In-House Beetles were evident corresponding to the different flock rotations (Table 2). The strongest indicator species correlated to In-House Beetle samples from Flock 1; *Jeotgalicoccus*, *Corynebacterium*, *Lactobacillus*, *Dietzia*, and *Aerococcus*. *Yaniella*, *Staphylococcus* and *Kluyvera* correlated with later flocks. The genus *Jeotgalicoccus* is within the Staphylococcaceae family and is considered to be widely distributed in nature. Species of *Jeotgalicoccus* have been previously found in poultry houses isolated from the feed supplements, *J. coquinae*, and the air filter system of a turkey house, *J. areolatus*, [19] and pig barns, *J. schoeneichii*, [20]. *Corynebacterium* is commonly found in the microbiota of animals, including poultry [21]. Bacteria from this genus exist mostly commensally, but some can be opportunistic pathogens, such as *C. diphtheriae*, the causative agent of diphtheria, which has been found in an outbreak in broiler chickens [22]. The pathogen *C. pseudotuberculosis* has been shown to be mechanically transmitted by house flies (*Musca domestica* L.), but no information exists about its association with this beetle species [23]. *Lactobacilli* are found in a variety of habitats, generally rich in carbohydrate substrates. This includes the mucosal, urogenital, and intestinal tracts of animals, and substances of plant origin, human and animal manure, and spoiling food waste. *Lactobacilli* produce propionic, acetic, and lactic acids that can result in reduced local pH, and potentially inhibit the growth of other bacteria [24]. *Lactobacilli* are also normal symbionts of invertebrates [25].

Vertebrates cannot synthesize carotenoids *de novo* and rely on dietary sources of intake, but the *Dietzia* spp. are well known for synthesizing carotenoids and have been used as a source of canthaxanthin [26]. Canthaxanthin (β, β-carotene-4,4′-dione) is a carotenoid, that has strong anti-oxidant activities and free radical scavenging properties, and thus has therapeutic use in diseases. Similar to *Rhodococcus*, is found in various habitats and is used as a supplement for animal feeding applications, such as aquaculture [27]. It has been found in poultry feed [28] and isolated in the midgut of mosquitoes [29]. *Yaniella* was originally isolated from soil, but has been reported in poultry litter [30,31,32]. Staphylococcus, a common environmental bacterium, is part of the normal flora of skin, intestines, and mucus membranes of animals including poultry [33]. Some species of *Staphylococcus* can cause Staphylococcosis, particularly in immunocompromised poultry, and if skin barriers are compromised by wounds allowing it to gain access to tissues and the bloodstream, systemic staphylococcal infections, such as septicemia and gangrenous dermatitis can result. It can be carried by various insects [34]. *Kluyvera* has been isolated from environmental samples, such as milk, water, soil, kitchen scraps and sewage, as well as the Eurasian spruce bark beetle, (*Ips typographus* L.), and can cause opportunistic bacteremia infections in immunocompromised individuals [35,36]. Due to its resemblance to Streptococcus, *Aerococcus* has only recently been recognized as a pathogen [37]. It has been isolated from both poultry and stored product insect pests, including flat grain beetles (*Cryptolestes* spp., Ganglbauer), lesser grain borers (*Rhyzopertha dominica*, Fabricius), and red flour beetles (*Tribolium castaneum*, Herbst) [38,39].

#### 3.1.3. In-House Beetle: Relative Abundances at the Phylum Level

The main phyla carried by the beetles includes the mostly thick peptidoglycan walled, thus Gram-positive, *Firmicutes* that are endospore producing bacteria consisting of two major groups. The groups are the Bacilli, a diverse obligate or facultative aerobe, and the anaerobic Clostridia that ferment gut carbohydrates to produce short chained fatty acids [40]. The Actinobacteria are mostly Gram-positive aerobic and usually found terrestrially in the soil, but can be aquatic as well, and perform a wide variety of metabolic actions vital to decomposition and humus formation [41]. Consequently, their helpful effects on the ecosystems within the soil have large economic importance; as well they generate antibiotic substances through their secondary metabolism. The Gram-negative Bacteroidetes have both anaerobia and aerobic non-spore forming species that are ubiquitous in the environment. Some are symbiotic species on the skin and in the gastrointestinal system of animals where they can degrade proteins or complex sugar polymers, a crucial function for their hosts. The Gram-negative Proteobacteria, are a very diverse group of bacteria and perform a wide variety of metabolic processes. This phylum includes a variety of foodborne pathogens important to the food production industry, notably *Salmonella* sp. and *Escherichia coli*. Additionally of note is the presence of a group of difficult to detect very small bacteria harbored by the beetles, the Tenericutes. Tenericutes are refractory to Gram staining because they are devoid of a peptidoglycan cell wall and tend to live intra- or intercellularly, as (endo)symbionts, parasites, pathogens, or commensals.

**Figure 1:**
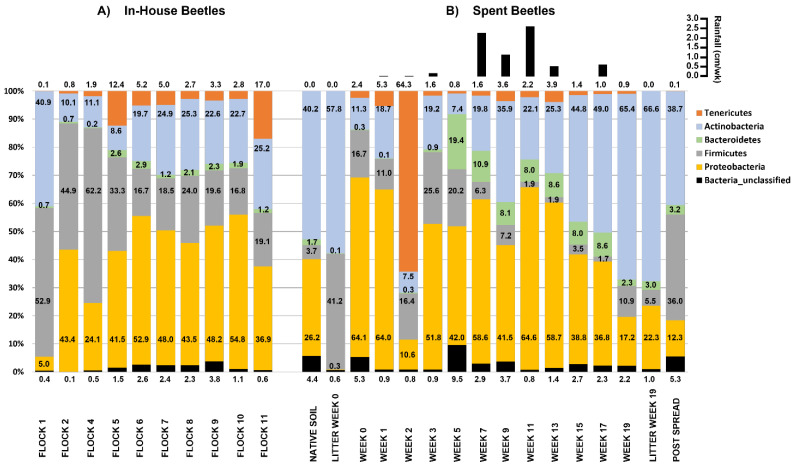
Histograms showing the mean relative abundances of bacterial phyla associated with (**A**) In-House Beetle samples taken during successive flock rotations, and (**B**) Spent Beetles taken after deposition of the litter into pastureland. The values correspond to the percentage of total sequences of that taxon for that sample. In the upper right-hand corner is weekly total rainfall (cm/wk) for weeks 1–3 and Post Spread, and bi-weekly total rainfall for weeks 3–19 at the site until the litter was spread on the field. *n* = 8 composite samples/Flock and 1 composite sample/Week.

Bedding and poultry were added to the house at the start of week 1 for the first flock rotation. LM were not found within the house until the 7th and final week of this first grow-out period (Flock 1). It is not known how they were introduced. Bacteria of phylum Firmicutes (53%) dominated the microbial community harbored by these first beetle invaders, followed closely by the Actinobacteria (41%), then Proteobacteria (5.0%), Bacteroidetes (0.7%), and Tenericutes (0.1%). This profile is similar to that of the litter in the first flock rotation prior to the beetles being found: Firmicutes (64%), Actinobacteria (30%), then Proteobacteria (5.6%), and Bacteroides (0.3%) [16]. As LM dwelt within the house that bacterial community structure rapidly changed, and the beetles sampled during Flock 2 showed a significant increase in Proteobacteria to 43% and a decrease in Actinobacteria to 10%. Beyond a fluctuation during Flock 4 and 5, the ratio (2.4:1) of Proteobacteria (mean = 47.4%) and Actinobacteria (mean = 19.1%) remained relatively stable during subsequent flock rotations (Flocks 6–11) (Figure 1A). Overall, the abundance of Firmicutes harbored by the beetles appreciably decreased over the first five successive flock rotations, seeming to stabilize at circa 23% by Flock 6 with Bacteroidetes averaging 1.9% of the microbial community in Flocks 6–11.

While our results represent both external and internal bacteria, Yun et al. [4] reported similar results after surveying the combined gut associated bacteria in 218 various species of insects with primers targeting the V1–V2 region of the 16S rRNA. They found that insect gut microbiota was dominated by Proteobacteria (62.1%) and Firmicutes (20.7%), followed by Bacteriodetes (6.4%), Actinobacteria (4.8%), Tenericutes (1.9%) and unclassified bacteria (3%). Using the V1–V3 targeting primers used in our present study, Wynants et al. [42] assessed the microbes in the larval stages from day 28 to 36 of LM and found a predominance of Proteobacteria (68–78%), followed by Firmicutes (~20%), then Actinobacteria which remained below 5% and Bacteriodetes at less than 1%. In comparison to the previous mentioned studies, the In-House Beetles carried more Actinobacteria, which could have resulted from their environment. From our previous work [16], we know that the In-House litter in which they dwelt, generally showed a consistent increase in the proportion of Actinobacteria and a decrease in Firmicutes over the 11 flock rotations, despite partial or total cleanouts. Additionally, the soil bacterial ecosystem under this litter, which the beetles inhabited, also showed an increase in Actinobacteria [43]. *Proteobacteria* showed a slow decline in the litter with a slight recovery after the partial and total cleanouts and the addition of new bedding [16]. Therefore, in comparison to the microorganisms in the litter, the LM carried more Proteobacteria, and less Actinobacteria and Firmicutes than their immediate environment.

The microbial diversity of LM has been studied as it relates to industrial production. Cucinin et al. [6] determined that LM reared on a factory protocol of soft wheat flour, corn flour, flaked corn, flaked whole barley, crushed broad beans, dried carob beans, and calcium carbonate (https://www.agripetgarden.it/ accessed on 9 December 2021) with carrots, carried six predominant phyla: Proteobacteria (57%), Bacteroidetes (26%), Firmicutes (8%), Actinobacteria (8%), Fusobacteria (<1%) and unclassified (<1%). This translated to eight classes of bacteria: Gammproteobacteria (57%) Bacteroidia (25%), *Bacilli* (7%) Actinobacteria (8%) Fusobacteria (1%) Alphaproteobacteria (<1%), Erysipelotrichia (<1%) and unclassified (<1%). In contrast the beetles in our study, found native to the poultry house averaged a lower percent abundance of Proteobacteria (41%), Bacteroidetes (1.6%), and Fusobacteria (<0.01%) and a higher percent of Firmicutes (32%), Actinobacteria (19%), Tenericutes (5%), and unclassified (1.5%). Additionally, the beetles in the spent litter over the 19 weeks averaged a lower percent abundance of Proteobacteria (37%), Bacteroidetes (6%), and *Fusobacteria* (<0.01%) and a higher percent of Actinobacteria (25%), Tenericutes (15%), Firmicutes (13%), and unclassified (3%). The stark taxa differences demonstrate the variability of bacteria with which LM can coexist.

#### 3.1.4. In-House Beetles: Beta Diversity & Analysis of Molecular Variance (AMOVA)

**Figure 2:**
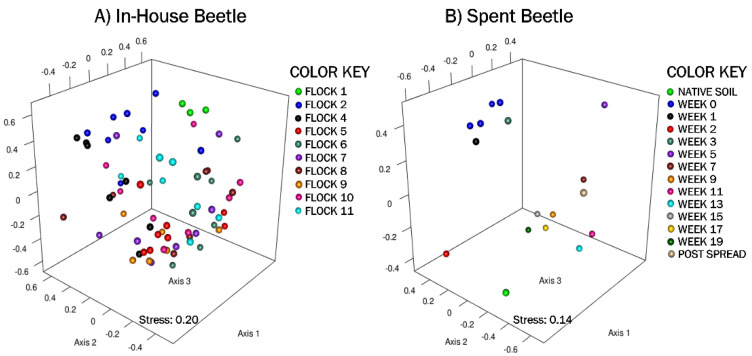
A non-metric multidimensional scaling (NMDS) ordination representing the bacterial community composition harbored by adult beetles, generated using Yue and Clayton distances, shows dissimilarities between components. Panel (**A**) shows clustering of the In-House Beetle samples (stress = 0.11, r^2^ = 0.92) over 11 flock rotations. Panel (**B**) shows clustering of the Spent Beetle samples (stress = 0.12, r^2^ = 0.89) over 20 weeks after piling in pastureland. *n* = 8 composite samples/Flock and 1 composite sample/Week.

The nonmetric multidimensional scaling (NMDS) plot, generated using Yue and Clayton genetic distances, showed very little dissimilarity patterns between components (Figure 2A). In general, there was overlap in the bacterial community structures among the beetles with the early separating only slightly from later flocks.

**Figure 3:**
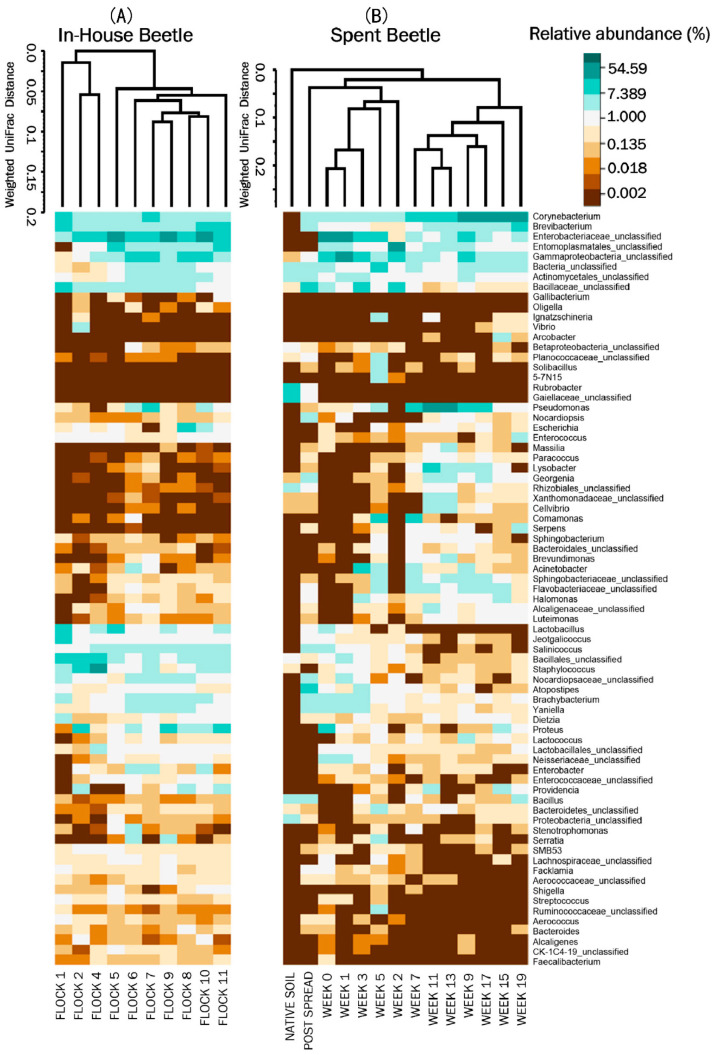
Heatmaps of the top 75 bacterial genera associated with LM during the (**A**) In-House Beetles associated with flock rotations; and (**B**) subsequent pastureland application of the Spent Beetle samples. In-House and Spent Beetle were clustered using Weighted Pair Group Method with Arithmetic Mean (UPGMA) tree based on weighted Unifrac distances. For natural log transformation “0” was converted to “0.01”. *n* = 8 composite samples/Flock and 1 composite sample/Week.

The weighted heat map of the genus level taxa harbored by the beetles showed shifts in microbial genera profiles between flock rotations (Figure 3A). AMOVA analyses of the genus level (*p* ≥ 0.01) parallels the UNIFRAC analyses, Flocks 1, 2 and 4 separate into their own clade as having different microbial communities from the subsequent 5 through 11 flock rotations. Flock 4 further separates into a clade with Flock 2. AMOVA analyses also demonstrates a difference in the LM microbial community in the earlier flocks versus the later flocks. Of the later flock rotations, LM from Flocks 5 and 11 separate out from Flocks 6 through 10 which had only minor fluctuations in community structure. The major management practice disruption of a partial (after Flock 7) or a total (after Flock 9) cleanout seemed not to disturb the microbial community harbored by the LM. The separation of early flocks (Flock 1 through 4) from the later flocks (Flock 5 through 11) was characterized by the increasing mean% abundance (>1% change) of unclassified *Enterobacteriaceae*, unclassified Entomoplasmatales, unclassified Gammaproteobacteria, *Pseudomonas*, unclassified Actinomycetales, *Escherichia*, *Yaniella*, *Salinicoccus*, *Brachybacterium*; and decreasing mean% abundance (>1% change) of genera *Staphylococcus*, *Corynebacterium*, *Jeotgalicoccus*, *Lactobacillus*, *Vibrio*, and *Brevibacterium* along with unclassified members of the family Bacillaceae and order Bacillales.

### 3.2. Spent Beetles

In this a newly constructed poultry house, the beetles first appeared near the end of the first flock rotation. We continued to collect the LM from the spent litter taken from the total cleanout of the house (after Flock 9) as they were relocated within the litter, which was piled into pastureland to be used as fertilizer. Arunraj and colleagues (2013) previously demonstrated that LM transported in poultry litter and placed in open fields remained alive and dispersed into the local environment up to 10 days later when the study ended. In the study presented here, litter beetles were found alive 19 weeks later until the poultry litter was spread on the pastureland. During that time, the LM were exposed to all of the biotic and abiotic environmental fluctuations common to the location, including local fauna, intermittent rainfall and changes in temperature.

#### 3.2.1. Spent Beetle: Alpha Diversity

The bacterial taxonomic diversity in the Spent Beetle samples was investigated (Table 3). Rarefaction curves are shown in Supplementary Materials Figure S1. The LM collected showed a large range in diversity over the weeks subsisting in the spent litter. The Inverse Simpsons at 0.01 genetic distance ranged from 2.4 during week 2, which showed a spike in Tenericutes and a decrease in Proteobacteria; to 34.3 during week 5, which showed a spike in unclassified bacteria and Bacteroidetes along with a decrease in Tenericutes and *Actinobacteria* from the previous weeks. In general, the microbial diversity was highest between weeks 3 through 13. This variability in diversity did not appear to correlate with rainfall or temperature changes at the site (Supplementary Materials Table S2); nor did it correlate with microbial diversity changes in litter samples taken from the site [16].

#### 3.2.2. Spent Beetle: Indicator Species

The Spent Beetle, after deposition onto the pastureland, had indicators of the fluctuations differentiating early (weeks 0–5) versus later (weeks 7–19) sampling, as the litter decomposed in the environment (Table 4). Some were previously discussed for the In-House Beetle indicator species section: *Yaniella*, *Lactobacillus*, and *Corneybacterium*. The bacteria *Brachybacterium* were actually first isolated from deep poultry litter and later classified as *Brachybacterium faecium* [44]. It is generally considered a harmless environmental bacterium and has been isolated with various insects [45,46]. The genus *SMB53* is a poorly described genus belonging to the family Clostridiaceae. Members of this family have been studied in the gut of earthworms (*Lumbricus terrestris* L.) for their ability to utilize glucose derived carbons [47]. *Salinicoccus* sp. Has been described in the gut microbiome of poultry as well as Mediterranean fruit flies (Ceratitis capitata, Weidemann) [48,49]. It has a bright pink-orange colored pigment which shows antimicrobial activity against other bacterial strains, including *Escherichia coli*, *Klebsiella pneumoniae*, *Bacillus subtilis*, *Proteus vulgaris*, *Pseudomonas aeruginosa*, and *Staphylococcus aureus* [50,51] and has shown the potential to degrade glyphosate [52].

*Luteimonas* is common in soil and marine sediments, but has been described in the gut of poultry and isolated from the wormcast of the earthworm (*Eisenia foetida*, Savigny) [49,53]. *Georgenia* is a thermotolerant bacteria that was shown to participate in organic matter degradation during the compositing of chicken manure [54]. *Pseudomonas* is a genus with 191 species demonstrating a wide array of metabolic capabilities that allows the colonization of diverse environments, mainly soil and aquatic environments, but it has been found in poultry microbiomes [49,55]. Some Pseudomonads are pathogens to various species and some, such as *P. fluorescens*, *P. luminescens* and *P. entomophila*, are able to produce insecticides [55]. *Paracoccus* is a diverse genus in which *Paracoccus marcusii*, like the *Dietzia* sp. discussed above, produces carotenoids studied for utilization as a source of pigmentation in layer hen feed to enhance egg yolk color [56,57]. *Paracoccus denitrificans* is studied industrially for its nitrate reducing capabilities and can cause the degradation of nitrogen additives used to fertilize crop soils. *Brevibacterium* also reduces nitrates to nitrites and has been isolated from poultry and poultry litter [31,49].

#### 3.2.3. Spent Beetle: Relative Abundances at Phylum Level

For each week after the poultry litter was piled in the pastureland, the relative abundance of bacteria harbored by the beetles fluctuated at the phylum level (Figure 1B). Similar to the In-House Beetles, the initial LM collected from the spent litter samples had a high proportion of *Proteobacteria* and a corresponding low proportion of *Actinobacteria*. However, over the weeks at the pastureland site, the proportion of *Proteobacteria* slowly decreased from 64.1% at week 1 to 17.2% of the microbial population at week 19. Conversely, the *Actinobacteria* slowly increased from 11.3% to 65.4%. *Firmicutes* populations fluctuated ranging from a high of 16.7% to a low of 1.7% over 19 weeks and Bacteriodetes ranged from 19.4% to 0.1%. Tenericutes were present at an average of 2.3% with the exclusion of week 2 in which Tenericutes represented 64% of the microbial population. The reason for this sudden spike in Tenericutes is unknown. Within the Tenericutes is the class *Mollicutes*, which is notable in that its genera tend to live intra- or intercellularly, and are (endo) symbionts, parasites, pathogens, or commensals. The order Entomoplasmatales that contains the family of helical Spiroplasmataceae consisting of the genus *Spiroplasma*, and the family non-helical Entomoplasmataceae consisting of two genera, the *Entomoplasma* and the *Mesoplasma*; are all associated primarily with the gut of arthropod hosts [58]. Although substantial weekly fluctuations occurred the overall mean change resulted in a decrease in Firmicutes (30.8 to 10.3%), whereas there were increases for Proteobacteria (39.8 to 45.7%), Actinobacteria (21.1 to 27.2%), Tenericutes (5.1 to 7.4%), Bacteroidetes (1.6 to 6.3%) and unclassified bacteria (1.5 to 2.8%). Unlike the changes in the microbial population in the litter which appeared to be influenced by rainfall, see Crippen et al. (2021), the microbial population associated by the adult LM did not appear influenced by the occurrence of rainfall. However, LM at the collection site were more readily located with the added moisture to the litter pile (personal observation).

#### 3.2.4. Spent Litter: Beta Diversity & AMOVA

The NMDS ordination of the Spent Beetle microbial composition shows some dissimilarities between components (Figure 2B). The native soil, and weeks 1, 2, 3 and 5 plotted furthest from the later weeks. The weighted heat map of the genus level taxa harbored by the LM corroborates shifts in microbial genera profiles between weeks in the pastureland (Figure 3B). The clustering analyses shows native soil the farthest distance from LM clustering into its own clade. In the next level clade, the Post Spread sample separates out, which consists mostly of soil with litter spread at 0.9 MT/0.4 ha. Therefore, the LM had little in common with the pastureland soil microbial community.

A comparison evaluating which bacteria varied over the weeks that the LM were in the pastureland was conducted. The LM of the early weeks (week 0 through 5) separated from LM of the later weeks (7 through 19) (Figure 3). The mean percent abundance (>1% change) changes of the major phyla were Proteobacteria (−1.3%), Actinobacteria (24.7%), Firmicutes (−13.2%), Tenericutes (−12.8%), Bacteroidetes (3.6%) and unclassified bacteria (−1.2%). The changes were characterized by the increasing mean% abundance in the later weeks of genera *Corynebacterium*, *Pseudomonas*, *Brevibacterium*, *Lysobacter*, *Georgenia*, *Providencia*, as well as unclassified members of the families Flavobacteriaceae, Sphingobacteriaceae, and Alcaligenaceae; and the decreasing mean% abundance (>1% change) of genera *Yaniella*, *Proteus*, *Brachybacterium*, and *Salinicoccus*, as well as unclassified members from families Enterobacteriaceae, Bacillaceae, Nocardiopsaceae, Neisseriaceae and order Entomoplasmatales.

**Figure 4:**
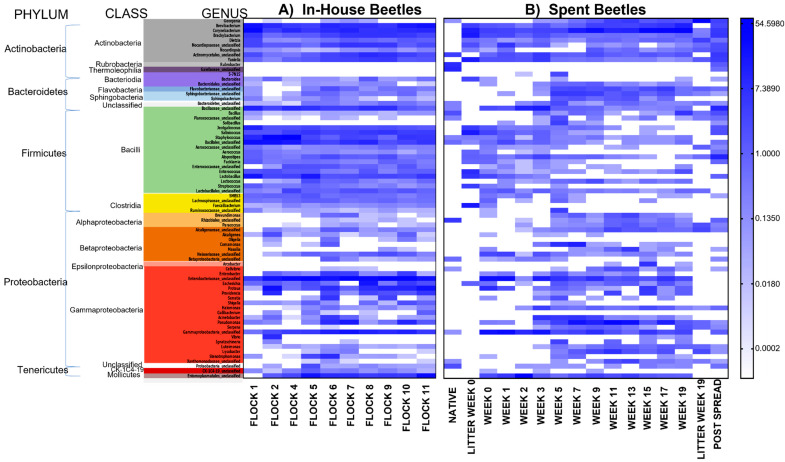
Heat map of natural log-transformed quantity of identified genera sequences (unweighted) in the LM beetles from the (**A**) In-House (grouped by class) collected during the flock rotations and (**B**) pastureland prior to deposition of the spent litter (native), samples of the Spent litter at the time of deposition into the pastureland and just prior to spreading (litter week 0 and litter week 19, respectively) and the soil/litter combination after spreading onto the pastureland (Post Spread); and the beetles collected from that litter over the 19 weeks of deposition (week 0 through week 19). *n* = 8 composite samples/Flock and 1 composite sample/Week.

A comparison evaluating which bacteria were reduced or not only retained, but augmented once the LM were moved from within the house to the pastureland was conducted. Such changes affect not just the bacterial community structure, but likely the metabolic activities of bacteria harbored by the LM. Of the mean% abundance of bacteria harbored by the In-House versus the Spent Beetles the genera *Corynebacterium*, *Pseudomonas*, *Comamonas*, *Georgenia*, and the family Sphingobacteriaceae bacteria increased the most; and the genera *Salinicoccus*, *Brevibacterium*, *Lactobacillus*, *Proteus*, *Staphylococcus*, unclassified members of family *Enterobacteriaceae*, and of orders the Entomoplasmatales and Bacillales decreased the most in the LM in the pastureland (Figure 4).

It is likely that the litter environment would have substantial influence on the LM microbial community. The outside environment can obviously influence the piled litter in which the LM reside, and the physiological properties of this pastureland litter was analyzed in a previous publication [8]. Briefly, analyses of the litter reported in our previous work, demonstrates that Ca, Cu, Fe, Mg, percent moisture, NO^3−^/N% organic matter, pH, P and Zn all increased; K and Mn increased only slightly, while electrical conductivity, Na, and S decreased in the litter over the 20 weeks it was piled in the pastureland. The litter likely influenced some of the bacteria harbored by the LM and reciprocally the beetles could have harbored bacteria that was exchanged with and affected the litter microbial profile, as demonstrated by Wynants et al. [42] that showed that most of the species-level operational taxonomic units, present in the feed substrate in which LM larvae were reared were also detected in the larvae. Our lab has demonstrated the exchange of bacterial pathogens between litter beetles and their environment [59]. Such a comparison can be done for the present data utilizing the litter microbial data already published [16] and evaluating which bacteria common to both beetles and litter that were augmented or reduced in the litter once it was deposited onto the pastureland. The comparison is shown in Figure 4, which includes the microbial profile of the litter at the time of deposition into the pastureland and just prior to spreading (litter week 0 and week 19, respectively). However, the variability in the microbial profile in environmental elements including in the soil can differ significantly just a few centimeters apart and presumably this is true in the litter environment as well; particularly due to abiotic stressors, such as water availability, oxygen concentrations, pH and carbon availability; which is why composite samples were made in this study [60]. Additionally, the density of beetles within the spent litter fluctuated greatly over the 20 weeks in pastureland and was much less dense than in the broiler house (personal observation), so to conclude any direct influence on the spent litter microbiome by the beetles is purely speculative and would require further study. With that caveat, an analysis of the most numerous bacteria carried by LM that were also present in the spent litter and showed an increase in the litter for the genera *Bacillus*, *Dietzia*, *Georgenia*, *Halomonas*, *Luteimonas*, *Lysobacter*, *Nocardiopsis*, *Paracoccus*, *Pseudomonas*, *Serpens* along with unclassified families Aerococcaceae, Alcaligenaceae, Flavobacteriaceae, Planococcaceae, Sphingobacteriaceae, Xanthomonadaceae, order Lactobacillales, and phylum Bacteriodetes between weeks 0 and 19 (Figure 4). Conversely, there was a decrease in the litter for the genera *Aerococcus*, *Atopostipes*, *Bacteroides*, *Brachybacterium*, *Brevibacterium*, *Corynebacterium*, *Enterococcus*, *Escherichia*, *Facklamia*, *Faecalibacterium*, *Jeotgalicoccus*, *Lactobacillus*, *Salinicoccus*, *Staphylococcus*, *Streptococcus*, *Yaniella*, along with unclassified members of the family Nocardiopsaceae, and the order Bacillales. *Rubrobacter*, *Gaiellaceae*_unclassified and 5–7N15 were present in In-House Beetles but not Spent Beetles and *Dysgonomonas*, *Delftia* and *Gallibacterium* present in Spent Beetles but not In-House Beetles.

In addition to the microbes of the LM present in the litter, one should also consider the potential contribution the LM make to the fertilizer through frass production. The growth in the commercial production of insects as a protein source has led to the assessment of the use of the mealworm frass, a byproduct resulting from that production, as a fertilizer. Recent studies of the frass of the closely related yellow mealworm (*Tenebrio molitor* L.) found it to be an effective boost in minerals when used as plant fertilizer to supply phosphorus and potassium irrespective of its application rate and nitrogen at 10t ha-1 application rates [61]. A microbiological study of the frass produced showed that the presence of microorganisms improved plant growth parameters by promoting high amounts of phosphorus, potassium, manganese, and magnesium, but that the microbial profile was dependent on the diet of the mealworm [62].

## 4. Conclusions

Overall, it is interesting and possibly concerning that the beetles were able to survive for so long in the litter piled in the open environment. These beetles carried an active microbiome which could presumably interact with the local environment and insectivores that might feed on the LM in the pastureland. Once in the open environment their microbial community began to change, particularly in the later weeks (after week 5), and showed major shifts in structure that did not cluster with the earlier weeks, the native soil, nor the post spread litter. The most abundant phyla harbored by the beetles while in the house were the Proteobacteria (39.8%), followed by the Firmicutes (30.8%), the Actinobacteria (21.1%), Tenericutes (5.1%), Bacteroidetes (1.6%) and unclassified bacteria (1.5%). However, over the time they resided in the outdoor environment their microbial profile underwent modifications characterized by a major decrease in the mean percent abundance (>1% change) of Firmicutes classes Clostridia and Bacilli (−20.5%); while Actinobacteria slightly increased (6.1%), as did Proteobacteria (5.9%), Bacteroidetes (4.7%), Tenericutes (2.3%), and unclassified bacteria (1.2%). The decrease in Clostridia and Bacilli could be advantageous from a risk standpoint, but dependent on the particular species the increase in some Gammaproteobacteria could be concerning. Their ability to survive for months in the pastureland within the spent litter and their risk as an in-house reservoir capable of transferring pathogens into the environment should be assessed. However, on the flip side the large amounts of frass excreted by these beetles could contain many minerals produced by beneficial microbial organisms that improve plant growth when the litter is used as a fertilizer.

## Figures and Tables

**Table 1:** The coverage, diversity, and evenness indices at 0.03 and 0.10 genetic distances of In-House Beetles bacteria associated with flock rotations. *n* = 8 composite samples/Flock.

	Coverage		Inverse Simpson Index		Shannon Index		Shannon Evenness Index	
In-House	0.03	0.1	0.03	0.1	0.03	0.1	0.03	0.1
Flock 1	0.94	0.97	18.66	11.41	3.53	3.00	0.79	0.74
Flock 2	0.95	0.97	8.57	5.51	2.36	1.87	0.59	0.53
Flock 4	0.94	0.97	9.07	5.50	2.78	2.33	0.65	0.60
Flock 5	0.92	0.95	20.08	11.64	3.50	3.00	0.77	0.71
Flock 6	0.89	0.94	18.22	11.13	3.44	2.97	0.74	0.70
Flock 7	0.90	0.95	15.18	8.42	3.44	2.88	0.75	0.68
Flock 8	0.90	0.95	18.17	11.59	3.48	3.00	0.74	0.70
Flock 9	0.90	0.95	16.24	8.43	3.40	2.77	0.73	0.65
Flock 10	0.90	0.95	13.81	7.41	3.34	2.57	0.72	0.64
Flock 11	0.92	0.96	11.01	6.58	3.06	2.52	0.68	0.63

**Table 2:** Indicator species for In-House Beetles sampled during flock rotations. Only those genera whose relative abundances across all flocks were 0.05% or higher and had a *p*-value of ≤ 0.001 and Point Biserial Correlation Coefficient (PBCC) * value ≥ 0.50 are shown. *n* = 8 composite samples/Flock.

Genera	Indicator Group	PBCC *
*Jeotgalicoccus*	Flock 1	0.839
*Corynebacterium*	Flock 1	0.748
*Lactobacillus*	Flocks 1, 5	0.667
*Dietzia*	Flock 1	0.663
*Yaniella*	Flocks 6, 7, 8, 9	0.660
*Staphylococcus*	Flocks 2, 4	0.649
*Kluyvera*	Flock 10	0.629
*Aerococcus*	Flock 1	0.618
Bacillaceae_unclassified	Flocks 1, 4, 5, 8, 9	0.588

* PBCC: Is a correlation between two binary vectors, similar to Indicator values. The higher the PBCC values the stronger the likelihood that a particular taxon is an indicator of that sample.

**Table 3:** The coverage, diversity, and evenness indices at 0.03 and 0.10 genetic distances of bacteria associated with *A. diaperinus* the weeks after the Spent litter was placed into the pastureland. *n* = 1 composite sample/Week.

	Coverage		Inverse Simpson Index		Shannon Index		Shannon Evenness Index	
Field	0.03	0.1	0.03	0.1	0.03	0.1	0.03	0.1
Week 0	0.92	0.96	9.77	4.79	3.10	2.42	0.69	0.60
Week 1	0.91	0.96	7.55	2.96	2.83	2.01	0.64	0.51
Week 2	0.93	0.96	2.38	2.36	1.90	1.77	0.45	0.45
Week 3	0.87	0.94	20.20	7.85	3.82	3.08	0.79	0.71
Week 5	0.64	0.79	74.89	34.32	5.00	4.33	0.89	0.83
Week 7	0.88	0.94	17.41	9.52	3.66	2.95	0.76	0.69
Week 9	0.88	0.95	14.30	10.09	3.63	2.99	0.76	0.72
Week 11	0.86	0.93	18.55	7.58	3.85	3.06	0.77	0.69
Week 13	0.88	0.94	23.88	10.49	3.91	3.21	0.80	0.73
Week 15	0.80	0.88	8.42	7.47	3.62	3.20	0.71	0.67
Week 17	0.86	0.93	7.07	5.98	3.34	2.75	0.68	0.63
Week 19	0.83	0.90	5.14	4.70	3.11	2.71	0.63	0.59

**Table 4:** Indicator species for Spent Beetle sampled while piled in pastureland. Samples were group in Weeks 0–5 and Weeks 7–19 for comparisons. Only those genera whose relative abundances across all weeks were 0.05% or higher and had a *p*-value ≤ 0.01 and Point Biserial Correlation Coefficient (PBCC) * value ≥ 0.50 are shown. *n* = 1 composite sample/Week.

Genera	Indicator Group	PBCC *	Genera	Indicator Group	PBCC *
*Brachybacterium*	Weeks 0–5	0.747	*Corynebacterium*	Weeks 7–19	0.786
*SMB53*	Weeks 0–5	0.715	*Luteimonas*	Weeks 7–19	0.747
*Yaniella*	Weeks 0–5	0.690	Gemm.3_unclassified	Weeks 7–19	0.732
Gammaproteobacteria_unclassified	Weeks 0–5	0.687	*Georgenia*	Weeks 7–19	0.729
Aerococcaceae_unclassified	Weeks 0–5	0.664	*Pseudomonas*	Weeks 7–19	0.683
*Salinicoccus*	Weeks 0–5	0.661	*Paracoccus*	Weeks 7–19	0.674
Betaproteobacteria_unclassified	Weeks 0–5	0.640	Rhizobiales_unclassified	Weeks 7–19	0.666
Bacillaceae_unclassified	Weeks 0–5	0.582	Alcaligenaceae_unclassified	Weeks 7–19	0.663
Bacillales_unclassified	Weeks 0–5	0.568	*Brevibacterium*	Weeks 7–19	0.609
*Lactobacillus*	Weeks 0–5	0.523			

* PBCC: Is a correlation between two binary vectors, similar to Indicator values. The higher the PBCC values the stronger the likelihood that a particular taxon is an indicator of that sample.

## Data Availability

All raw sequence files were submitted to European Nucleotide Archive Database as part of this study (accession# PRJEB47980).

## Supplementary Materials

The following are available online at https://www.mdpi.com/article/10.3390/microorganisms10010175/s1, Figure S1: Schematic of the broiler facility; Figure S2: Rarefaction Curves; Table S1: The Feeding Regime; Table S2: The Mean Weekly Environmental Conditions.
