# Supplementary material for: Adult Alphitobius diaperinus Microbial Community during Broiler Production and in Spent Litter after Stockpiling
Source: Microorganisms. 2022 Jan 14;10(1):175. doi: 10.3390/microorganisms10010175 (PMC8778262; doi:10.3390/microorganisms10010175)
Supplement: Supplementary file 1 [file microorganisms-10-00175-s001.zip › Table S2.pdf]

Table S2. The mean weekly environmental conditions at the site where the Spent litter was piled.

Week	T (°C)[a]	Range T (°C)	Rainfall (cm)[b]	Humidity (%)[a]	Wind Speed (kph)[a]
1	18	23-12	0.02	50	9
2	19	25-14	0.07	57	3
3	19	24-14	0.18	58	6
4	21	28-13	0.05	55	7
5	22	31-14	0.00	53	6
6	24	29-20	2.05	64	10
7	26	31-21	0.47	68	6
8	27	33-21	0.00	67	4
9	26	31-21	1.27	68	3
10	27	31-24	2.90	74	6
11	29	34-24	0.00	73	6
12	28	33-23	0.05	72	4
13	28	32-23	0.53	73	5
14	28	33-24	0.00	73	4
15	29	34-24	0.00	72	6
16	29	35-24	0.00	73	4
17	29	34-23	0.65	71	3
18	30	37-23	0.00	69	3
19	32	37-24	0.00	74	4
20	32	39-26	0.00	67	4

[a] Mean weekly temperature (T), humidity and wind speed.

[b] Total rainfall for the week.
