# Supplementary figures and images for: Adult Alphitobius diaperinus Microbial Community during Broiler Production and in Spent Litter after Stockpiling
Source: Microorganisms. 2022 Jan 14;10(1):175. doi: 10.3390/microorganisms10010175 (PMC8778262; doi:10.3390/microorganisms10010175)

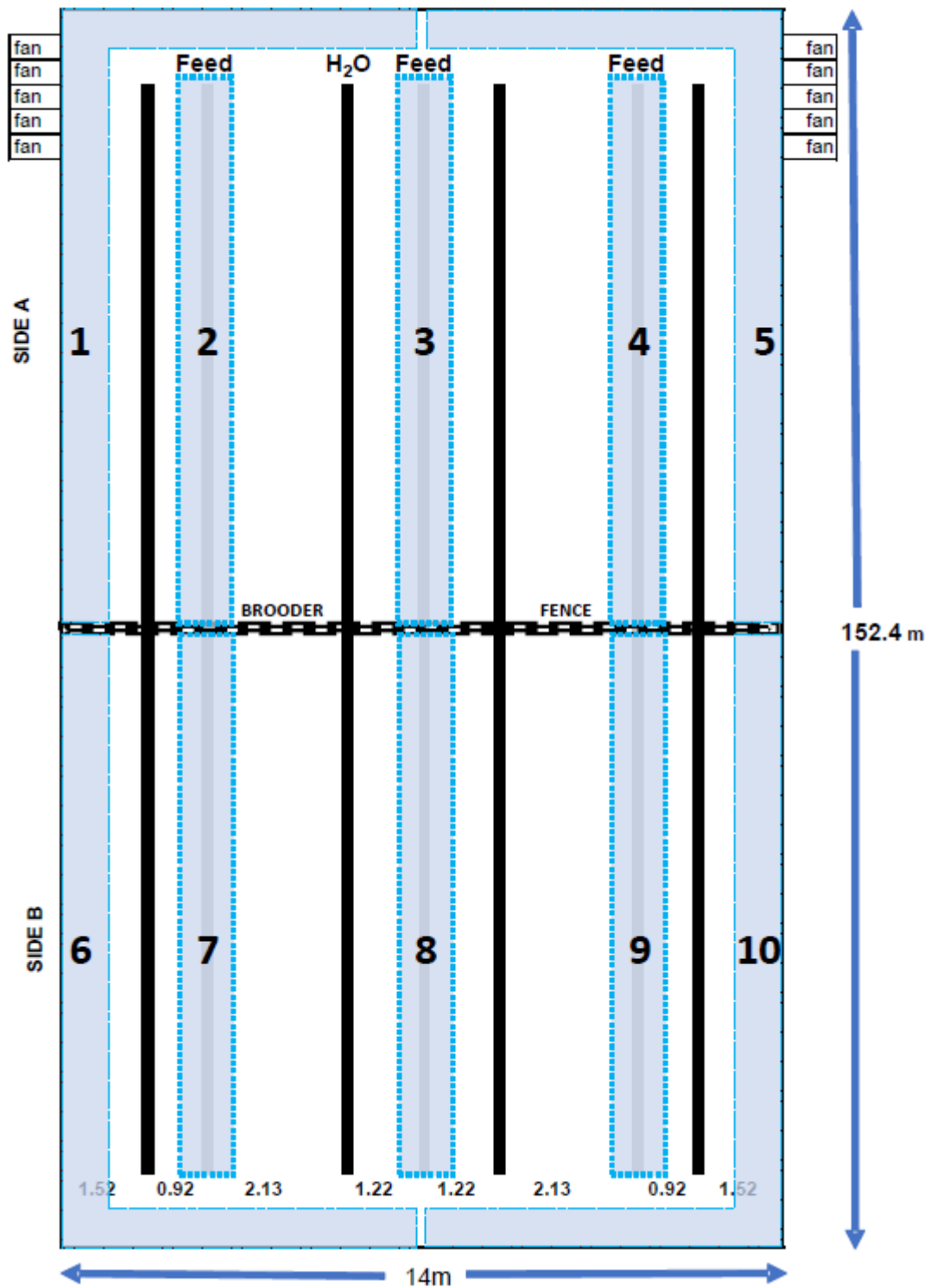

Figure S1: Schematic of the broiler facility.

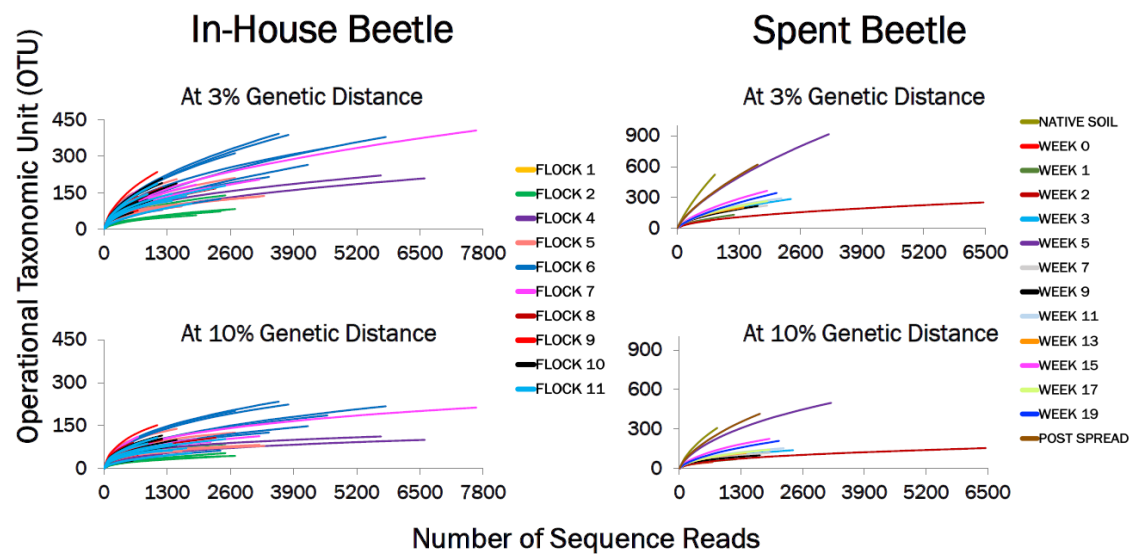

Figure S2: Rarefaction Curves.

Supplement: Supplementary file 1 [file microorganisms-10-00175-s001.zip › microorganisms-1521983-supplementary.pdf]
